# 

**Published:** 1897-12-18

**Authors:** 


					iuu hospital, ABSOLUTELY PURE, therefore BEST. Dec. isth, 1897.
CADBURY'S ww w CADBURY'S
OOCOA Irl OOCOA
' The standard of highest purity at present ML /MK
attainable.*'?Lancet, NO ALKALIES USED,
Ci "  ^ ~ '^NORFOLK ah* NORWICH-HOSPLtAL
No. 586.' Vol. XXIII. [a&Taper!] SATURDAY,
Deo. 18th, 1897.
[SubBcnpfonTerms,] pRICE TWOPENCE.

				

